# Looking for New Herbal Treatments for Metabolic Syndrome

**DOI:** 10.1155/2016/7198490

**Published:** 2016-06-01

**Authors:** Hyeung-Jin Jang, Jae-Young Um, Hanseok Ko, Won-Seok Chung

**Affiliations:** ^1^Department of Biochemistry, College of Korean Medicine, Kyung Hee University, Seoul 02447, Republic of Korea; ^2^Department of Pharmacology, College of Korean Medicine, Kyung Hee University, Seoul 02447, Republic of Korea; ^3^Neuroregeneration and Stem Cell Programs, Institute for Cell Engineering, The Johns Hopkins University School of Medicine, Baltimore, MD 21205, USA; ^4^Department of Korean Rehabilitation Medicine, College of Korean Medicine, Kyung Hee University, Seoul 02447, Republic of Korea

Diabetes, which is one of the metabolic diseases, is a complex and chronic illness to keep maintaining medical and diet care against abnormal glycemic symptoms such as high blood glucose level and insulin resistance. This diabetes, which is inextricably bound up with obesity, can cause decrease of life expectancy, increase of healthcare cost, and weakening of quality of life by risk of acute or long-term complication. In particular, type 2 diabetes mellitus (T2DM) rate of all diabetes reported about over 90%. Also, prevailing population and increasing healthcare budget of diabetes are conservatively estimated to be as much as 366 million people and be as much as 396 billion international dollars in 2030. Even if many clinical doctors and researchers have developed medical care and found therapeutic agent for T2DM, it is difficult to avoid side effects such as hypoglycemia, hyperinsulinemia, diarrhea, and heart failure. Here, this special issue suggested how we accessed alternative treatment or safe therapy for T2DM patients by introducing several research papers using herbal medicines.

Traditional medicine has a history of more than 3000 years in clinical trials including China and Korea. The major use of herbal medicines is for health promotion about diverse disease including T2DM and therapy for chronic, as opposed to life-threatening, conditions. It is the most important reason for which herbal medicines are widely perceived as natural and safe without side effects. The historical factors that the herbal medicine has been prescribed in T2DM patients and evaluated safety against side effects for a long time let our group experimentally test whether hyperglycemia effects by taking herbal medicines are related to glucagon-like peptide-1 (GLP-1) stimulation via taste receptor signaling on enteroendocrine L cells or not. Firstly, our group tested whether prescribed extracts of herbal medicines,* Citrus aurantium, Anemarrhena asphodeloides, *and* Bupleurum falcatum, *were responsible for GLP-1 secretion and taste receptor signaling or not in enteroendocrine L cells using GLP-1 assay and microarray. GLP-1 is one of the incretin hormones and is secreted from intestinal enteroendocrine L cells, which existed mainly in the proximal ileum and colon, and GLP-1 is induced through nutrient sensing taste receptor that responds to sweet, bitter, umami, and fatty acids. These herbal medicines stimulated GLP-1 secretion and upregulated the mRNA of taste receptor genes in L cells. Additionally, we tested whether GLP-1 secretion practically affected hyperglycemia effects on diabetic mice model or not. Diabetic mice exhibited more ameliorated hyperglycemia than control group. However, this GLP-1 secretion by herbal medicines lacks biochemical evidence. It is unknown which components on herbal medicines stimulate GLP-1 and which specific taste receptor signaling among sweet, bitter, umami, and fatty acids was activated by herbal medicines. To overcome these limitations, the study supplemented systemic inhibition study and component analysis on herbal medicine,* Gentiana scabra *extracts. In aspect of systemic inhibition study, pharmacologic inhibitors associated with taste receptor signaling on GLP-1 and calcium imaging study and knockout mice of *α*-gustducin applied to mechanism definition of taste receptor signaling of* Gentiana scabra *extracts on* in vivo* study of plasma GLP-1 and insulin measurements. Furthermore, to confirm components in* Gentiana scabra *extracts and to evaluate active compound of GLP-1 secretion, the components of* Gentiana scabra* extractswere analyzed by HPLC-MS and active compound of GLP-1 secretion in L cells was selected by GLP-1 assay.

This issue tried to explain safe application for T2DM patients by regulating hyperglycemia via taste receptor signaling. We provide previous study of therapeutic application via taste receptor signaling for T2DM patients and challenging limitation such as structural assessments. To accurately define specific taste receptor of binding difference according to chemical characters or structure, the components derived from herbal medicines of structural assessment binding taste receptor should be reinforced to cooperate with structural biology areas. Also, for GLP-1 secretagogue of discovery of evidence-based complementary and alternative medicine via taste receptor signaling, development of agonistic component with taste receptor from each herbal medicine, structural assessment of specific taste receptor on discovery agonistic component, and its GLP-1 secretagogue of clinical evaluation need to be supported by future researches.

*Hyeung-Jin Jang* *Hyeung-Jin Jang* *Jae-Young Um* *Jae-Young Um* *Hanseok Ko* *Hanseok Ko* *Won-Seok Chung* *Won-Seok Chung*

